# Cholecystitis with abdominal wall biloma after percutaneous transhepatic gallbladder aspiration: A case report

**DOI:** 10.1016/j.ijscr.2020.09.108

**Published:** 2020-09-18

**Authors:** Kenichiro Toritani, Mitsutaka Sugita, Akiko Shimizu, Akira Watanabe, Hidetaka Andrew Ono, Hiroyuki Baba

**Affiliations:** Department of Surgery, Yokohama City Minato Red Cross Hospital, Yokohma, Japan

**Keywords:** PTGBA, percutaneous transhepatic gallbladder aspiration, CRP, C-reactive protein level, CT, computed tomography, PTGBD, percutaneous transhepatic gallbladder drainage, Abdominal wall biloma, Case report, Cholecystitis, PTGBA

## Abstract

**Background:**

Abdominal wall biloma is an uncommon entity. We herein report an extremely rare case of cholecystitis with abdominal wall biloma after percutaneous transhepatic gallbladder aspiration (PTGBA).

**Presentation of case:**

A 69-year-old woman was diagnosed with acute cholecystitis, and PTGBA was performed on Day 1. PTGBA was performed a second time because of re-expansion of the gallbladder and an increased CRP level on Day 3. Computed tomography was performed on Day 9 because we suspected recurrence of cholecystitis. It revealed a well-circumscribed fluid collection between the abdominal wall or the diaphragm and the liver. Based on these intraoperative findings, we diagnosed her with cholecystitis with abdominal wall biloma. Cholecystectomy and drainage of the abdominal wall biloma were performed on Day 10. The postoperative course was uneventful, and she was discharged on Day 18.

**Discussion:**

Early cholecystectomy is the gold-standard treatment for acute cholecystitis, but cholecystectomy is not performed in some cases. PTGBA is much more convenient, quicker, and less costly, but inappropriate aspiration during the second PTGBA session might have spread the infected bile to the abdominal wall through the PTGBA route.

**Conclusion:**

This case represents the first reported case of a biloma within the abdominal wall after PTGBA. To prevent this complication, we should aspirate gallbladder bile sufficiently during PTGBA. In addition, we should consider performing alternative therapy, such as percutaneous transhepatic gallbladder drainage or an operation, when we fail to appropriately aspirate.

## Highlights

• The complication of percutaneous transhepatic gallbladder aspiration.
• Inappropriate aspiration may cause an abdominal wall biloma.
• We should consider alternative therapy when aspiration was not sufficiently.

## 1 Background

The term “biloma”, denoting an encapsulated extrahepatic collection of bile, was first introduced by Gould and Patel in 1979 [1]. A biloma mainly has an iatrogenic or traumatic cause and is often located at the subcapsular space of the right hepatic lobe or intra-abdominal space, being rarely located at the abdominal wall.

This is the first report of a case of a biloma within the abdominal wall after percutaneous transhepatic gallbladder aspiration (PTGBA). This case report has been reported in line with the SCARE checklist [2].

## 2 Presentation of case

**Fig. 1:**
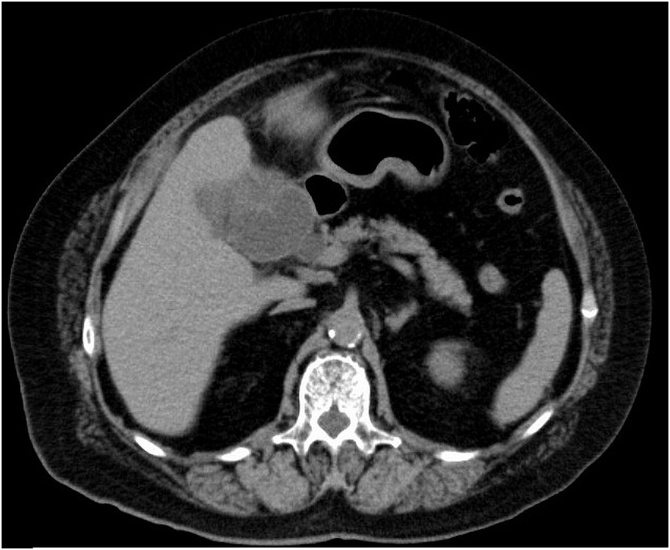
Image from abdominal enhanced computed tomography (CT) before PTGBA. CT revealed an enlarged gallbladder. There was no fluid collection.

A 69-year-old woman was admitted to our hospital at night due to acute upper abdominal pain. She was known to suffer from hypertension. An abdominal examination showed tenderness in the right upper quadrant. Murphy’s sign was positive. Initial investigations showed an elevated white blood cell number (18400/μl) and C-reactive protein level (CRP; 4.8 mg/dl). Renal and liver function tests and amylase and coagulation screens were normal, as were her chest and abdominal plain findings. Computed tomography (CT) revealed enlargement of the gallbladder with a thickened and enhanced wall. There was a gallstone in the gallbladder. There was no fluid collection around the gallbladder (Fig. 1). We diagnosed this case as acute cholecystitis Grade 2, so we considered early cholecystectomy according to the Tokyo guideline 2018 [3,4]. But cholecystectomy could not be performed because of shortage of operation staff in midnight. So, PTGBA was performed on Day 1.

**Fig. 2:**
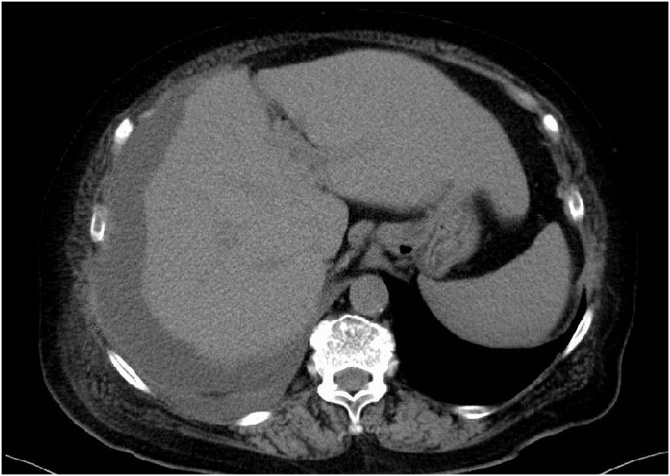
Image from abdominal enhanced computed tomography (CT) after the second PTGBA. CT revealed a large well-circumscribed fluid collection between abdominal wall, diaphragm and liver.

PTGBA was carried out with a 21-gauge needle under sonographic guidance. Infected bile (80 mL) was aspirated, and the needle was removed immediately. Because of re-expansion of the gallbladder and a further increased CRP level (35 mg/dl), PTGBA was performed a second time on Day 3, and 45 mL of infected bile was aspirated. She began to recover temporarily, but her abdominal pain and high fever recurred on Day 9. CT revealed a large, well-circumscribed fluid collection between the right diaphragm and the liver (Fig. 2).

**Fig. 3:**
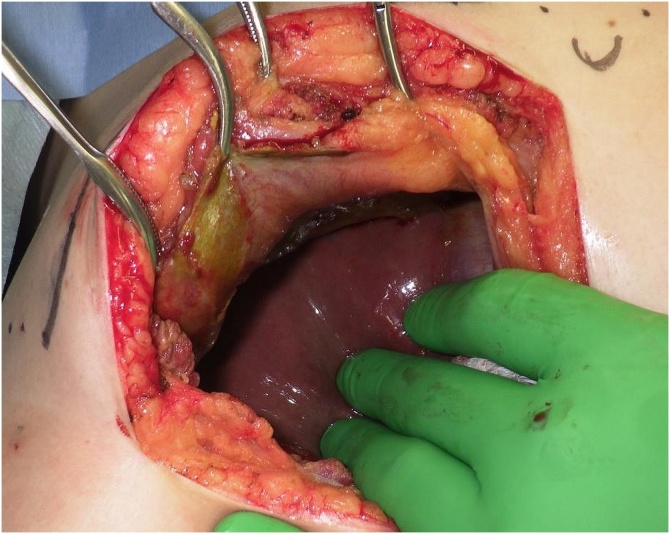

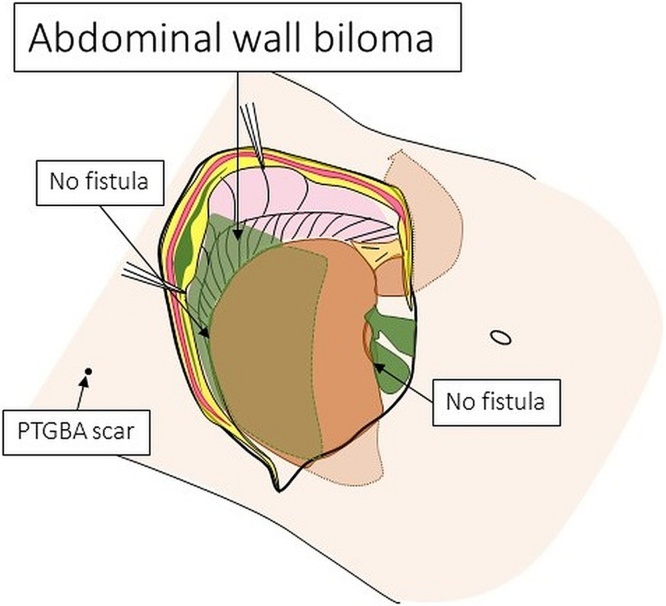
A, B. Images from Operative findings. Abdominal wall biloma was located between parietal peritoneum and abdominal lateral muscle, diaphragm. A; Operative image. B; Schema of operative finding.

We planned open cholecystectomy for cholecystitis, and the drainage of subcapsular biloma of the liver on Day 10. Regarding the intraoperative findings, there is no bile and infectious in peritoneal cavity. The gallbladder was tense and reddened, but no perforation or fistula with the abdominal wall was seen. The liver was normal, and no fistula was noted between the liver and the abdominal wall, nor was a subcapsular biloma of the liver detected. There was an abdominal wall biloma located between the parietal peritoneum and abdominal lateral muscle or diaphragm (Fig. 3A-B). We performed cholecystectomy. The biliary system appeared normal on intraoperative cholangiography. We also performed fenestration and curettage of the biloma as much as possible. A 19-Fr Blake drain was placed under the right subphrenic space for drainage of the residual biloma. Drainage was performed until postoperative Day 5, and antibiotic therapy was performed until postoperative Day 6. She was discharged home on postoperative Day 9 without any complications.

## 3 Discussion

Early cholecystectomy is the gold-standard treatment for acute cholecystitis [3,4]. In principle, the treatment strategy on our institute for acute cholecystitis is according to the Tokyo guideline 2018. But cholecystectomy cannot be performed in some cases because of hospitals lack the capacity to perform the operation, e.g. too few operation rooms or shortage of operation staff. In such cases, an alternative treatment, such as PTGBA or percutaneous transhepatic gallbladder drainage (PTGBD), is performed. PTGBA is much more convenient and quicker than PTGBD, so even for cases encountered in the middle of the night, PTGBA can be performed with just a few staff members at the bedside in the emergency room with only a needle and an ultrasound sector transducer.

A previous study also showed that PTGBA was safer than PTGBD [5]. Although a randomized control trial revealed that PTGBD was superior to PTGBA in terms of clinical effectiveness [6], another study reported that repetitive procedures improved the clinical effectiveness of PTGBA [7]. Another study showed that improvement of clinical symptoms was obtained in 60 % of cases with a one-time PTGBA procedure and in 88 % of cases with a second PTGBA procedure [8]. Because of patient's desire to avoid semi-urgent cholecystectomy, we performed repetitive PTGBAs in this case, but we experienced a rare complication of an abdominal wall biloma.

Bilomas, which are encapsulated, extrahepatic collections of bile, are often located in the subcapsular liver and intra-abdominal space. Abdominal wall biloma is extremely rare. There have only been two case reports of abdominal wall biloma, but both were complications of cholecystectomy [9,10]. The precise mechanism underlying the formation of abdominal wall biloma after PTGBA is unclear. Previous papers have described the cause of abdominal wall biloma as iatrogenic, with bile leaking from the gallbladder bed or from a small accessory duct tracking along the drainage tube and collecting within the abdominal wall [9,10].

In the present case, inadequate aspiration during the second PTGBA might have caused the spreading of infected bile to the abdominal wall through the route of PTGBA, as the amount of bile aspirated during the second PTGBA was less than that of the first, and the gallbladder was extremely tense on intraoperative findings. Intrahepatic biliary tract injury by PTGBA was denied by intraoperative cholangiography, and there was no fistula between the gallbladder, liver and abdominal wall.

To prevent a biloma from developing within the abdominal wall after PTGBA, we should aspirate the gallbladder bile sufficiently during PTGBA. When we are unable to aspirate sufficiently during PTGBA, we should consider alternative therapy, such as PTGBD or emergency cholecystectomy.

The pre-operative diagnosis of abdominal wall biloma is difficult. In the present case, CT revealed a well-circumscribed right subdiaphragmatic collection. We diagnosed her with subscapular biloma of the liver preoperatively because it sometimes occurs with acute cholecystitis [[11], [12], [13]] and there was no report of abdominal wall biloma after PTGBA.

## 4 Conclusions

We presented our experience with an extremely rare case of cholecystitis with abdominal wall biloma after PTGBA. To prevent this complication, we should aspirate gallbladder bile sufficiently during PTGBA. In addition, we should consider alternative therapy, such as PTGBD or emergency cholecystectomy, when we fail to aspirate sufficiently during PTGBA.

## Declaration of Competing Interest

The authors report no declarations of interest.

## Funding

All authors declare no source of funding.

## Ethical approval

The clinical case is exempt from ethical approval.

## Consent

Informed consent signed by a patient to publish the article.

## Author’s contribution

Kenichiro Toritani: wrote the paper, study concept or design, data collection, data analysis and interpretation, performed the surgery.

Mitsutaka Sugita: contribute to interpretation, revision, data collection and data analysis.

Akiko Shimizu: performed the surgery.

Akira Watanabe: approved the paper.

Hidetaka Andrew Ono: approved the paper.

Hiroyuki Baba: performed the surgery.

All authors have approved the final article for submission.

## Guarantor

Kenichiro Toritani.

## Provenance and peer review

Not commissioned, externally peer-reviewed.
